# A novel photo-regulated self-healing hydrogel based on hollow SiO_2_@g-C_3_N_4_@TiO_2_ and PVA†

**DOI:** 10.1039/d5ra01507c

**Published:** 2025-06-23

**Authors:** Yu-Ling Qi, Hao-Yu Zhou, Guo-Zhi Han

**Affiliations:** a College of Chemistry and Molecular Engineering, Nanjing Tech University Nanjing 211816 P. R. China han@njtech.edu.cn

## Abstract

Self-healing hydrogels have the ability to repair themselves at the incision after being damaged and can return to their original state of morphology and performance. However, constructing a hydrogel with superior mechanical strength and tensile properties after self-healing remains a challenge. In this work, using polyvinyl alcohol, borax, chitosan, and a type of SiO_2_@g-C_3_N_4_@TiO_2_ nanoparticles as raw materials, a novel photo-regulated self-healing hydrogel was developed using a freezing–thawing method, which could achieve a synchronous increase in Young's modulus and tensile strength under visible light irradiation during the self-healing process. In addition, the doping of the SiO_2_@g-C_3_N_4_@TiO_2_ nanoparticles improved the self-healing performance of the PVA-based hydrogels. With the addition of a trace amount of SiO_2_@g-C_3_N_4_@TiO_2_ nanoparticles, the self-healing efficiency of the hydrogel increased from 26.67% to 45.67% in darkness and from 41.33% to 65.67% under visible light irradiation.

## Abstract 2

A novel kind of photo-regulated self-healing hydrogel was developed by freezing–thawing method. The synchronous growth of Young's modulus, tensile strength and self-healing performance were achieved by visible light irradiation.

## Graphical abstract

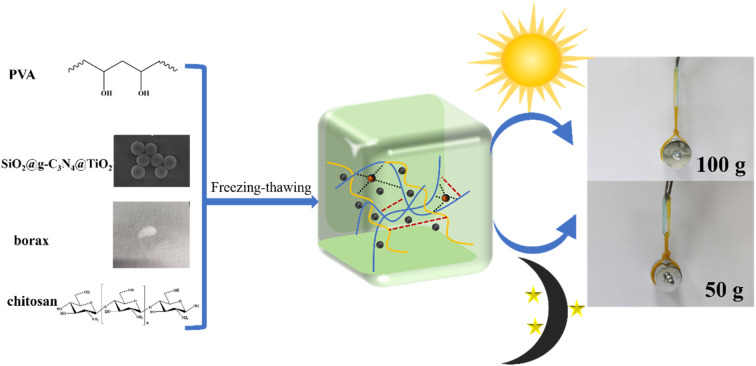

## 1. Introduction

Functional materials often suffer from some external injuries during use, affecting the performance of the materials and resulting in waste. Therefore, materials with self-healing properties have emerged as required by the times. Self-healing refers to the ability of self-repairing after external injuries. Through self-healing, the service life of the materials can be extended, and the cost of materials can be greatly reduced.^1–3^ In the past decade, due to the wide-ranging applications in human tissue engineering,^4–6^ drug delivery^7–9^ and flexible bionic materials,^10–12^ hydrogels with self-healing ability have become a research hotspot in the field of functional materials. Hydrogels usually refer to polymer materials with a three-dimensional network structure containing a large number of hydrophilic groups such as –OH, –CONH^−^ and –COOH, which endow the materials with a strong water retention capacity.^13–15^ The common design idea for self-healing hydrogels is to introduce reversible cross-linking between polymers in the hydrogels. According to different cross-linking mechanisms, self-healing hydrogels can be divided into two categories: chemical self-healing hydrogels and physical self-healing hydrogels.^16,17^ Chemical self-healing hydrogels achieve self-healing through dynamic covalent bonds or reversible chemical reactions, and physical self-healing hydrogels achieve self-healing through dynamic non-covalent bonds.^18–21^ The dynamic covalent bond refers to a type of covalent bond that can break or exchange reversibly under external stimulation.^22,23^ Unlike traditional covalent bonds, the dynamic covalent bonds have the advantages of stimulus response and reversibility. Physical self-healing hydrogels achieve self-healing usually without external stimulation through dynamic non-covalent linking, mainly including hydrogen bonding,^24,25^ host–guest interactions,^26–30^ π–π stacking^31,32^ and hydrophobic associations.^33,34^

Currently, hydrogels based on biofriendly macromolecules have been widely used in electronic devices,^35,36^ drug delivery,^37–39^ wastewater treatment,^40–42^ tissue engineering,^43–45^*etc.* Among these hydrogels, polyvinyl alcohol (PVA) has attracted great attention owing to its high hydrophilicity, low toxicity, outstanding biodegradability, high water retention rate and good mechanical properties by hydrogen bonds. In particular, it was found that pristine PVA-based hydrogels possess a certain self-healing capacity, which meets the great account of both self-healing and biocompatibility in biomedical applications.^46–48^ Generally, PVA-based hydrogels were prepared using a freezing–thawing method. However, most pristine PVA hydrogels have the disadvantages of insufficient toughness and tensile strength after self-healing. To solve the problem, a common strategy was to introduce other inorganic or organic functional materials to construct composite hydrogels, thus improving the physical and mechanical properties of the PVA-based hydrogels.^49–52^ For example, using borax as a cross-linker and PVA, sodium alginate (SA) and tannic acid (TA) as raw materials, Zhao *et al.*^53^ prepared a multifunctional conductive composite hydrogel by a one-pot method, in which a type of double network was formed by borate ester bonds and hydrogen bonds. The obtained hydrogel exhibited high stretchability (780% strain) and rapid self-healing properties, and the healing efficiency (HE) was as high as 93.56% without any external stimulation. However, after self-healing for many times, the mechanical strength of hydrogel was often greatly reduced, which affects its application life in the actual process.

**Fig. 1:**
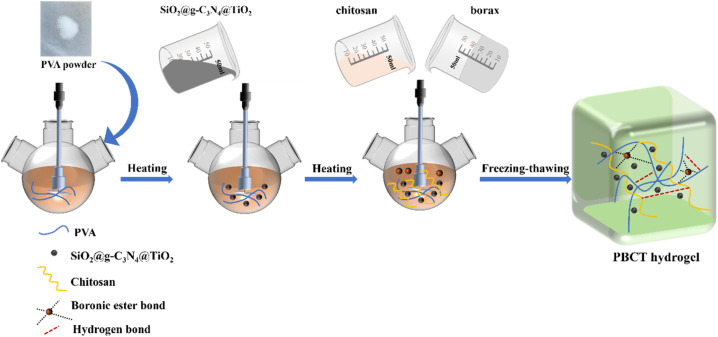
Preparation route of the PBCT hydrogel.

In recent years, the development of nanotechnology in the hydrogel field has entered a new stage. Due to the special physical and chemical effects, the introduction of nanomaterials would greatly improve the performance of traditional hydrogels.^54,55^ For example, for the application of hydrogels in biomedicine, the composite of semiconductor nanomaterials such as TiO_2_ has gradually entered the vision of the scientific community because of the anti-aging and anti-bacterial ability of the semiconductor nanomaterials.^56^ However, there are few studies on the effect of semiconductor nanomaterials on the self-healing properties of hydrogels. There is no doubt that the research on the issue is essential for the construction of organic/inorganic composite self-healing hydrogels. In this work, based on our previous work,^57^ using PVA, borax, chitosan, and a type of nanocomposite, SiO_2_@g-C_3_N_4_@TiO_2_, as raw materials, we developed a type of photo-regulated PVA-based hydrogel (PBCT) by a freezing–thawing method. The self-healing performance and mechanical properties of the composite hydrogel can be controlled by irradiation of light. More importantly, the synchronous increase in Young's modulus and tensile strength was realized by visible light irradiation in the self-healing process. The synthetic route of the hydrogel is shown in Fig. 1.

## 2. Experimental section

### 2.1. Materials and instruments

Polyvinyl alcohol (PVA, *M*_w_: ∼145 000 g mol^−1^) was obtained from Meryer Biochemical Technology Co., Ltd (Shanghai, China). Chitosan (CS, medium viscosity: 200–400 mPa s) and borax (sodium tetraborate decahydrate, Na_2_B_4_O_7_·10H_2_O) were purchased from Aladdin Industrial Corporation (Shanghai, China). All chemical reagents were of analytical grade and used without any further purification. The composite semiconductor nanoparticles of black SiO_2_@g-C_3_N_4_@TiO_2_ were self-prepared in our laboratory according to our previous work,^57^ which has uniform hollow microsphere morphology and photocatalytic performance, as shown in Fig. S1 and S2.† Fig. S3† shows the photoluminescence spectroscopy (PL) of the black SiO_2_@g-C_3_N_4_@TiO_2_ nanoparticles, which confirmed that generation of photo-generated electrons. The self-made deionized (DI) water was used in all experiments.

Scanning electron microscopic (SEM) images were obtained using a Jeiss Ultra Plus SEM microscope (Carl Zeiss, Germany). Transmission electron microscopic (TEM) observation was performed using a JEM-2100 (JEOL, Japan). The FT-IR spectra were recorded using a Nicolet AVATA (Thermo Fisher, USA). Thermogravimetric (TG) profiles were recorded using a TG209F3 thermogravimetric analyzer (Netzsch, Germany). X-ray diffraction (XRD) patterns were recorded using a D8 ADVANCE X-ray diffractometer (Bruker, Germany). The PL spectrum of sample was detected using an F97 Pro fluorescence spectrophotometer (SH Lingguang, China). The tests of electron paramagnetic resonance (EPR) were conducted using an EMXplus (Bruker, Germany). X-ray photoelectron spectra (XPS) were recorded using an AXIS SUPRA X-ray photoelectron spectrometer (Kratos, Japan). The UV-vis spectroscopy tests were conducted using a UV-3600 spectrophotometer (Shimadzu, Japan). Mechanical property data were obtained using a DY-LDW 50N electronic universal testing machine (Guangzhou Deyi testing equipment Co., Ltd, China). The light source for the self-healing process was a BBZM-I scientific research xenon lamp (BoBei Optical factory, China).

### 2.2. Preparation of the PBCT hydrogels

The PBCT hydrogels were synthesized by a one-pot method. First, 5 g PVA powder was added into 20 mL of deionized water in a 100 mL beaker, followed by continuous stirring at 95 °C for 4 h until PVA was completely dissolved to form a transparent homogenous solution (20 wt%). At the same time, a certain amount of SiO_2_@g-C_3_N_4_@TiO_2_ nanoparticles were dispersed in deionized water to form a dispersion solution (0.02 wt%). Moreover, 0.06 M borax aqueous solution and 1.4 × 10^−4^ M CS solution were prepared for use. Afterwards, a certain volume of the SiO_2_@g-C_3_N_4_@TiO_2_ dispersion solution, borax solution and CS solution were dropwise added to the PVA solution in succession. The mixed solution was continuously stirred at 95 °C until a steady SiO_2_@g-C_3_N_4_@TiO_2_/PVA/CS/borax mixed solution was formed. Finally, the samples were frozen at −25 °C for 12 h, followed by thawing at 25 °C for 10 h to form the PBCT hydrogels.

Without doubt, the amount of the SiO_2_@g-C_3_N_4_@TiO_2_ nanoparticles would affect the structure and property of the composite hydrogels. Therefore, we conducted a series of parallel optimization experiments as shown in Table 1. In this process, the total volume of the system was kept consistent by adjusting the amount of deionized water.

**Table 1:** Compositions of the PBCT hydrogel

Sample	PVA (g)	DI water (mL)	CS (mL)	Borax (mL)	SiO_2_@g-C_3_N_4_@TiO_2_ (mL)
PBCT-0	5.00	23.00	1.00	2.00	0.00
PBCT-1	5.00	22.00	1.00	2.00	1.00
PBCT-3	5.00	20.00	1.00	2.00	3.00
PBCT-5	5.00	18.00	1.00	2.00	5.00
PBCT-8	5.00	15.00	1.00	2.00	8.00

### 2.3. Mechanical properties tests

The hydrogel samples were prepared in a dumbbell shape with dimensions of 50 mm (length) × 7 mm (width) × 5 mm (thickness) for tensile testing at a loading rate of 100 mm min^−1^. The stress (*σ*) was obtained by dividing the loading force (*F*) by the cross-sectional area (*A*). The strain (*ε*) was obtained by dividing the deformation height (Δ*h*) by the original height (*h*). In the linear region of the stress–strain curve, Young's modulus was calculated as *E* = *σ*/*ε*. All experiments were conducted three times in parallel, and all the mechanical measurements were conducted in air condition. A xenon light was used to simulate the visible light.

### 2.4. Self-healing tests

To test the self-healing ability, the PBCT hydrogels were fabricated into a dumbbell-type shape and dyed with different colors in the preparation process. One sample of the hydrogel was stained with methyl orange, and the other sample was stained with methylene blue. Subsequently, the PBCT hydrogels were cut in half, and then the fresh cross-sections dyed with different colors were immediately put together along the fracture surfaces at room temperature. For investigating the effect of the SiO_2_@g-C_3_N_4_@TiO_2_ nanoparticles on the self-healing performance, a sample was irradiated by visible light in the self-healing process, and the other sample was allowed to stand in darkness. In order to maintain the consistency of the environment, the two samples were placed in the same room, as shown in Fig. S4.† After a certain time, the self-healing properties of the hydrogels were evaluated based on the detailed mechanical test. The healing efficiency was calculated using eqn (1) as follows:

1 HE (%) = *σ*_h_/*σ*_0_ × 100%

where *σ*_0_ and *σ*_h_ are the tensile stresses of the original and self-healing hydrogel samples, respectively. In addition, during the test, a small amount of glycerin was coated on the surface of the samples to prevent the impact of water loss caused by thermal effects.

### 2.5. Swelling ratio tests

Swelling ratio is an important index of hydrogels for practical applications. To investigate the effect of the SiO_2_@g-C_3_N_4_@TiO_2_ nanoparticles on the swelling performance of the hydrogels, the prepared PBCT hydrogels were dried at 50 °C for 4 h and then cut in square samples of dimensions 5 mm (width) × 5 mm (thickness). The mass of the samples was weighed and recorded as *M*_d_, and then, the hydrogel samples were put into 5 mL of deionized water at room temperature. After a certain time interval, the swelled hydrogels were taken out, and subsequently weighed after the water on the surface was wiped gently with a paper towel. Finally, the swelling ratios of the hydrogels were calculated using eqn (2):

2 SR (%) = (*M*_s_ − *M*_d_)/*M*_d_ × 100%

where *M*_s_ is the weight of the swollen samples and *M*_d_ is the weight of the dried samples.

## 3. Results and discussion

### 3.1. Structural characterization

**Fig. 2:**
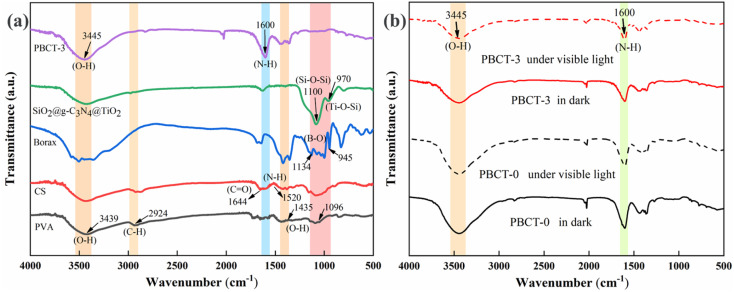
(a) FT-IR spectra of PVA, CS, borax, SiO_2_@g-C_3_N_4_@TiO_2_ and PBCT-3 hydrogels. (b) FT-IR spectra of PBCT-0/PBCT-3 hydrogels before and after light irradiation.

Fig. 2(a) shows the FT-IR spectra of PVA, CS, borax, SiO_2_@g-C_3_N_4_@TiO_2_ nanoparticles and the PBCT-3 hydrogel. The FT-IR spectra of PVA showed the following absorption bands: the broad bands at 3439, 1435, 1096 cm^−1^ were indexed to the stretching vibration of O–H, and the peak at 2924 cm^−1^ was assigned to the stretching vibration of C–H bonds. The FT-IR spectra of borax presented a strong absorption band between 945 and 1134 cm^−1^ corresponding to the B–O bond.^58^ As for the CS, peaks that appeared at 1644 cm^−1^ and 1520 cm^−1^ were attributed to the stretching vibration of C=O and N–H, respectively^.^^59^ In the FT-IR spectrum of SiO_2_@g-C_3_N_4_@TiO_2_ nanoparticles, the typical peak at 1100 cm^−1^ was indexed to the stretching vibration of Si–O–Si, and the peak at 970 cm^−1^ was assigned to the stretching vibration of Ti–O–Si.^57^ However, after the four materials were mixed to form the composite PBCT hydrogels, the characteristic peaks of SiO_2_@g-C_3_N_4_@TiO_2_ nanoparticles and borax were almost invisible, which may be due to that the content of the two components in the composite hydrogel was lower and covered by the PVA. Fig. 2(b) presents the FT-IR spectra of the PBCT-0 hydrogel and PBCT-3 hydrogel before and after visible light irradiation. A wide absorption band at 3445 cm^−1^ was observed in all samples, which were assigned to the stretching vibration of O–H and N–H bonds. In addition, no significant difference was observed in the spectra of the PBCT-0 hydrogel before and after light irradiation. However, for the PBCT-3 hydrogel, the above-mentioned peaks were somewhat attenuated after light irradiation, which may be due to the photochemical cross-linking catalyzed by the SiO_2_@g-C_3_N_4_@TiO_2_ nanoparticles.^60^

**Fig. 3:**
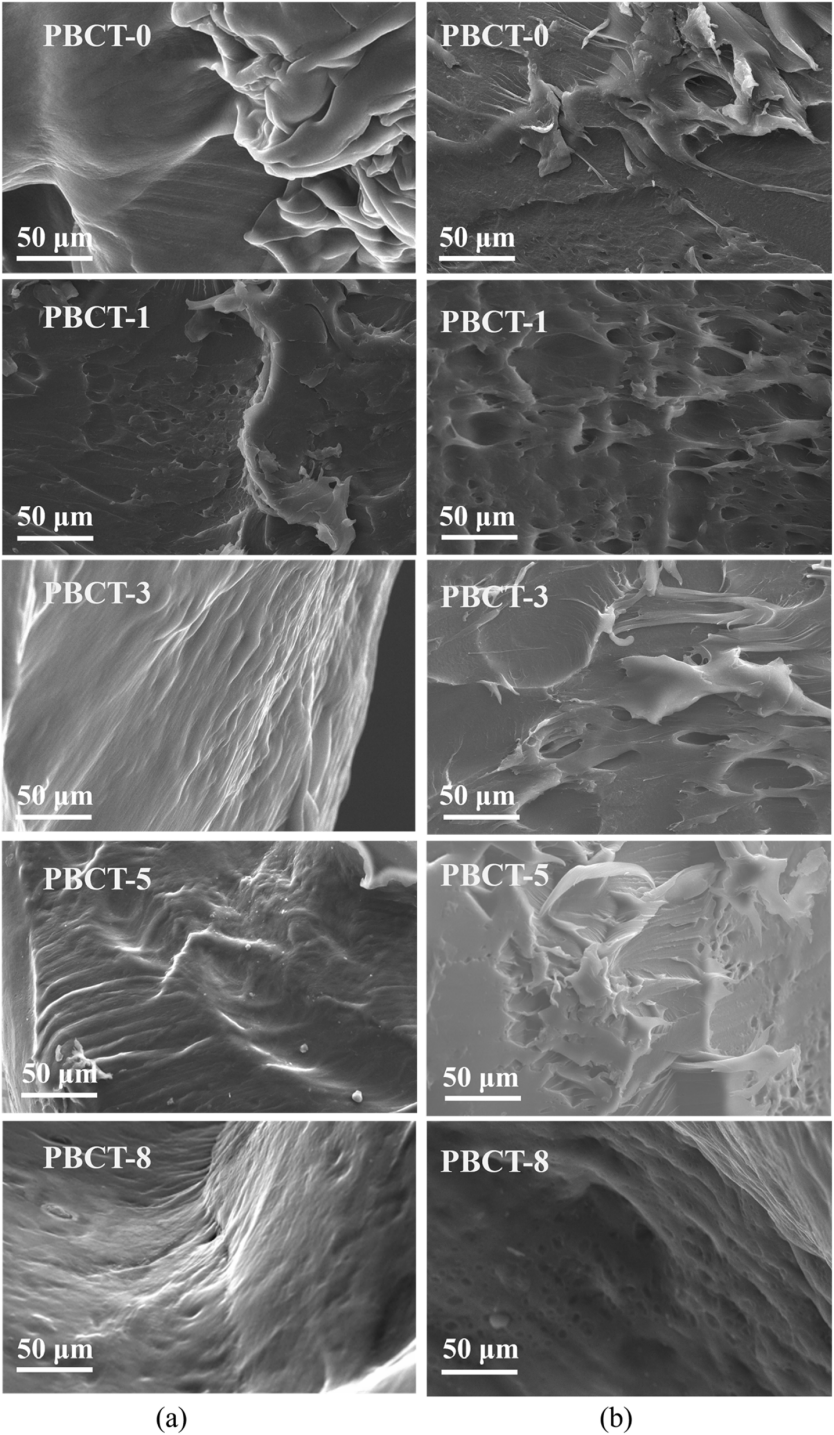
SEM images of the PBCT hydrogel before (a) and after (b) light irradiation.

Fig. 3 shows the SEM images of the self-healing section of the composite hydrogels with different amounts of SiO_2_@g-C_3_N_4_@TiO_2_ nanoparticles before and after visible light irradiation. The experimental results indicated that with the increase in nanoparticle content, the interface became smooth. This is because the abundant groups on the surface of the SiO_2_@g-C_3_N_4_@TiO_2_ nanoparticles promote the crosslinking of hydrogel molecules. Moreover, after light irradiation, the roughness of the self-healing section of the PBCT-0 hydrogel almost remained unchanged, but that of PBCT-1 and PBCT-3 significantly increased, as well as the increase of holes. This phenomenon may be due to the role of free radical active species produced in the light irradiation process, which promoted the crosslinking of hydrogel molecules and resulted in the shrinkage of polymer chains. Without doubt, the porous structure of the healing interface is conducive to improving the mechanical properties of the hydrogels. However, with the further increase in the nanoparticle dose, the porous structures of PBCT-5 and PBCT-8 were conversely weakened and changed little before and after visible light irradiation. This may be due to that too much SiO_2_@g-C_3_N_4_@TiO_2_ nanoparticles caused excessive cross-linking between polymer chains in the hydrogels.

**Fig. 4:**
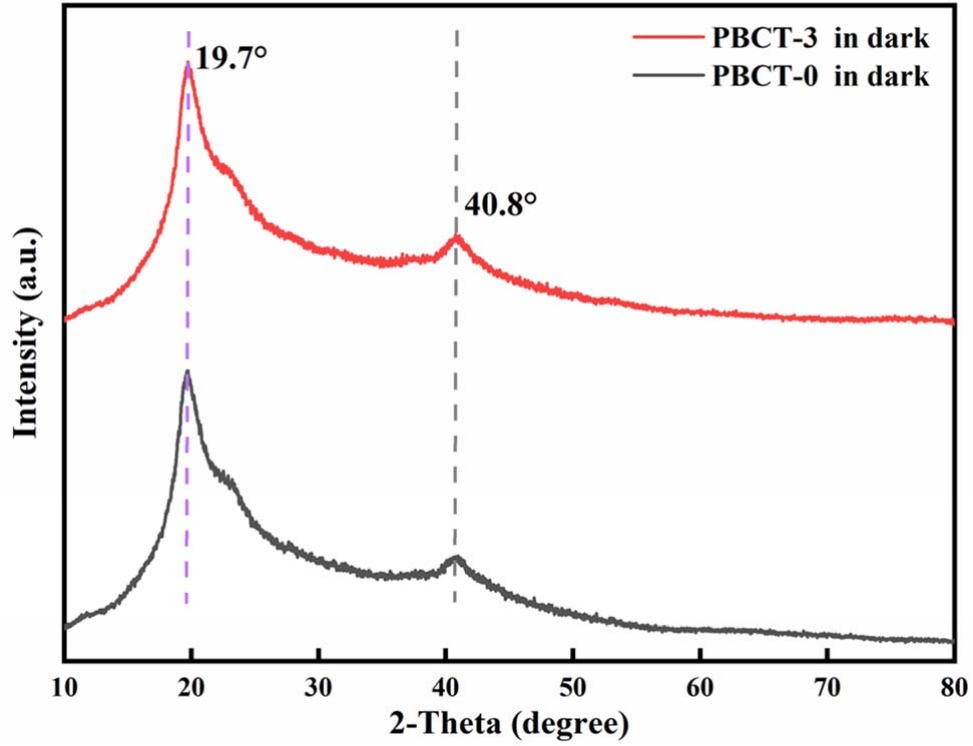
XRD patterns of PBCT-0 and PBCT-3 hydrogels.

Fig. 4 shows the XRD patterns of the composite hydrogels of PBCT-0 and PBCT-3. The characteristic diffraction peaks of neat PVA at 19.7° and 40.8° corresponded to the orthorhombic lattice structure of PVA microcrystals.^61^ Moreover, the diffraction peaks of SiO_2_@g-C_3_N_4_@TiO_2_ were not observed in the sample of PBCT-3, which should be masked by the PVA. Furthermore, the XRD patterns of PBCT-0 and PBCT-3 were very similar, indicating that the addition of a small amount of SiO_2_@g-C_3_N_4_@TiO_2_ nanoparticles did not significantly affect the crystallization behavior of polymers in the composite hydrogels.

**Fig. 5:**
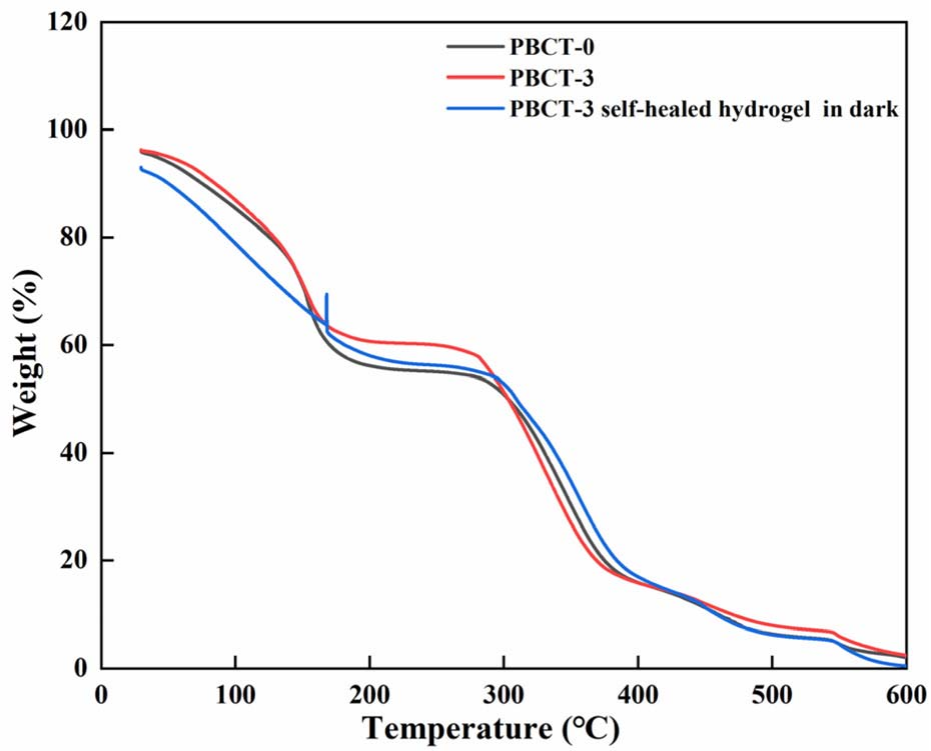
TG curves of the self-healed PBCT hydrogel.

Fig. 5 shows the impact of SiO_2_@g-C_3_N_4_@TiO_2_ nanoparticles on the thermal stability of the PBCT hydrogels. The experimental results showed that the mass loss of the composite hydrogels can be roughly divided into three stages. The mass loss of the first stage between 20 and 120 °C should be indexed to the evaporation of water in the composite hydrogels, and that of the second stage between 200 °C and 300 °C was probably caused by the decomposition of chitosan. The mass loss of the third stage between 300 °C and 600 °C was attributed to the complete decomposition of polymers. However, after mixing with the SiO_2_@g-C_3_N_4_@TiO_2_ nanoparticles, the mass loss of the composite hydrogel in the second stage was decreased. It may be due to that the SiO_2_@g-C_3_N_4_@TiO_2_ nanoparticles enhanced the molecules link of the CS in the composite hydrogels. Furthermore, after light irradiation, the mass loss of the PBCT-3 further decreased. The phenomenon was possible due to the photo-oxidative coupling of amino groups in CS catalyzed by the SiO_2_@g-C_3_N_4_@TiO_2_ nanoparticles,^62^ which was consistent with the previous FT-IR spectra. After the three stages, it was obviously seen that the final mass loss of the PBCT-3 hydrogel after irradiation was the smallest. The above-mentioned results confirmed that the photo-oxidation based on the semiconductor nanoparticles can improve the thermal stability of the composite hydrogel.

**Fig. 6:**
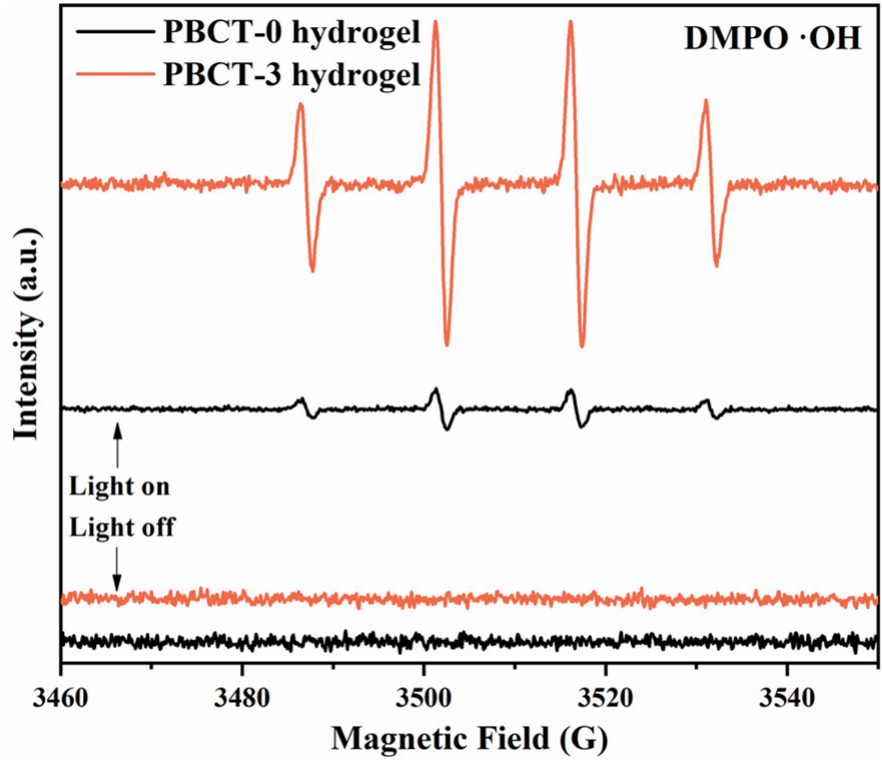
EPR spectra of PBCT-0 and PBCT-3 samples.

To validate the above-mentioned hypothesis, we further employed DMPO as a spin-trapping agent to carry out the electron paramagnetic resonance (EPR) tests of PBCT-0 and PBCT-3 samples, as shown in Fig. 6. Under the light irradiation, the PBCT-3 sample exhibited distinct signals of hydroxyl radicals (˙OH), displaying a characteristic quartet peak with a relative intensity ratio of 1/2/2/1. Notably, for the PBCT-0 sample, weak signals were also detected, which may be attributed to the trace decomposition of water molecules under light exposure.

Fig. S5† shows the UV-vis diffuse reflectance spectra of PBCT-0 and PBCT-5. It was obviously found that after the addition of the nanoparticles, the absorption of the visible light by the hydrogel was significantly enhanced, which confirmed the distribution of the nanoparticles in the PBCT hydrogels. Fig. S6† shows the XPS spectra of the PBCT-3 hydrogel under visible light irradiation and in darkness. As for the C 1s spectra, three peaks at 284.5 eV, 286.1 eV and 287.9 eV were indexed to C–C/C–H, C–O and O–C–O in PVA and CS, respectively. After visible light irradiation, the binding energy of C–O increased from 286.1 eV to 286.4 eV, and the binding energy of C–C/C–H increased from 284.5 eV to 284.7 eV, whereas the binding energy of O–C–O increased from 287.9 eV to 288.3 eV. Moreover, the proportion of C–O significantly decreased. In the spectra of N1s, three peaks at 397.5 eV, 399.3 eV and 401.3 eV were assigned to C=N–C, –NH_2_ and N–C=O in CS, respectively. After visible light irradiation, the binding energy of –NH_2_ increased from 399.3 eV to 399.7 eV. The binding energy of N–C=O increased from 401.3 eV to 401.7 eV, and the binding energy of C=N–C increased from 397.5 eV to 397.9 eV. It was worth noting that, the proportion of C=N–C was very small before light irradiation, whereas increased dramatically after light irradiation. Based on the above-mentioned results, we infer that under light irradiation, some hydroxyl groups of PVA or CS were oxidized to aldehyde groups, and subsequently, chemically coupled with amino groups of CS to form imines.

### 3.2. Self-healing properties of the composite PBCT hydrogels

**Fig. 7:**
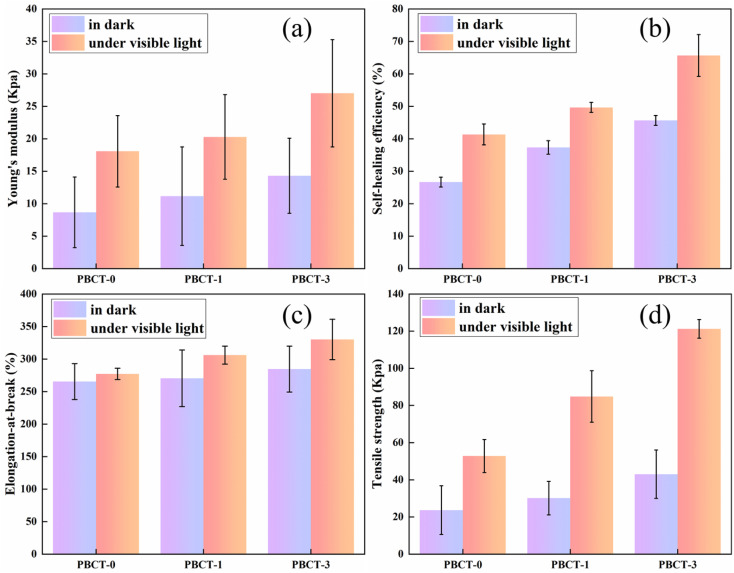
Effect of visible light irradiation on the self-healing of the PBCT hydrogels ((a) Young's modulus, (b) self-healing efficiency, (c) elongation-at-break, (d) tensile strength).

In order to explore the effect of the SiO_2_@g-C_3_N_4_@TiO_2_ nanoparticles on the self-healing performance of the composite hydrogels, the tensile-stress tests of the three types of composite hydrogels after self-healing were carried out, as shown in Fig. 7 and S7.†Fig. 7(a) shows that the SiO_2_@g-C_3_N_4_@TiO_2_ nanoparticles can effectively enhance Young's modulus of the composite hydrogel after self-healing. Without the SiO_2_@g-C_3_N_4_@TiO_2_ nanoparticles, Young's modulus of the hydrogel sample of PBCT-0 only reached 8.66 kPa after self-healing under room conditions. However, when a small amount of SiO_2_@g-C_3_N_4_@TiO_2_ nanoparticles was mixed, Young's moduli of PBCT-1 and PBCT-3 hydrogels slightly increased from 8.66 kPa to 11.16 kPa and 14.30 kPa, respectively. It implied that the SiO_2_@g-C_3_N_4_@TiO_2_ nanoparticles can enhance the interaction between polymers in the PBCT hydrogels, which should be attributed to the abundant hydroxyl groups on the surface of the SiO_2_@g-C_3_N_4_@TiO_2_ nanoparticles. Interestingly, if the self-healing process is accompanied by visible light irradiation, Young's modulus of PBCT-1 increased greatly from 11.16 kPa to 20.28 kPa, whereas that of the PBCT-3 hydrogel increased from 14.30 kPa to 27.01 kPa. As for the PBCT-0 hydrogel, Young's modulus also increased from 8.66 kPa to 18.07 kPa, which may be due to the loss of water cause by the thermal effect of light. This phenomenon indicated that light further promotes the crosslinking between polymers, which was consistent with the thermal stability test. Fig. 7(b) showed the self-healing efficiency of the PBCT hydrogels under different conditions. Experimental results indicated that the addition of a small amount of the SiO_2_@g-C_3_N_4_@TiO_2_ nanoparticles can greatly improve the self-healing efficiency of the hydrogel. Under visible light irradiation, the self-healing efficiency of the PBCT-0 increased from 26.67% to 41.33%, whereas that of the PBCT-1 increased from 37.33% to 49.67% and that of PBCT-3 increased from 45.67% to 65.67%. Fig. 7(c) shows the effect of visible light irradiation on the stretchability of the PBCT hydrogel after self-healing. Experimental results indicated that visible light irradiation effectively improves the stretchability of hydrogels containing the SiO_2_@g-C_3_N_4_@TiO_2_ nanoparticles after self-healing. The elongation-at-break of self-healed PBCT-0 had little changes with and without light irradiation. However, with visible light irradiation, the elongation-at-break of self-healed PBCT-1 increased from 270.49% to 306.13% in contrast to those in darkness, whereas that of self-healed PBCT-3 increased from 284.58% to 330.13% in contrast to those in darkness. Fig. 7(d) shows the effect of light irradiation on the tensile strength of the PBCT hydrogel after self-healing. With visible light irradiation, the tensile strength of the self-healed PBCT-0 increased from 23.67 kPa to 52.80 kPa, whereas that of self-healed PBCT-1 and PBCT-3 increased from 30.15 kPa to 84.85 kPa and from 43.03 kPa to 121.25 kPa, respectively. These results indicated that the addition of small amount of the SiO_2_@g-C_3_N_4_@TiO_2_ nanoparticles can greatly improve the mechanical properties of PBCT hydrogels after self-healing.

Next, in order to investigate the performance limits of the SiO_2_@g-C_3_N_4_@TiO_2_ nanoparticles in the composite hydrogels, we further prepared samples with 5 mL and 8 mL of SiO_2_@g-C_3_N_4_@TiO_2_ dispersion solution by the same method. The experimental results indicated that compared with PBCT-3, the mechanical performances and self-healing efficiency of PBCT-5 and PBCT-8 showed a certain degree of decrease, as shown in Fig. S8.†

**Fig. 8:**
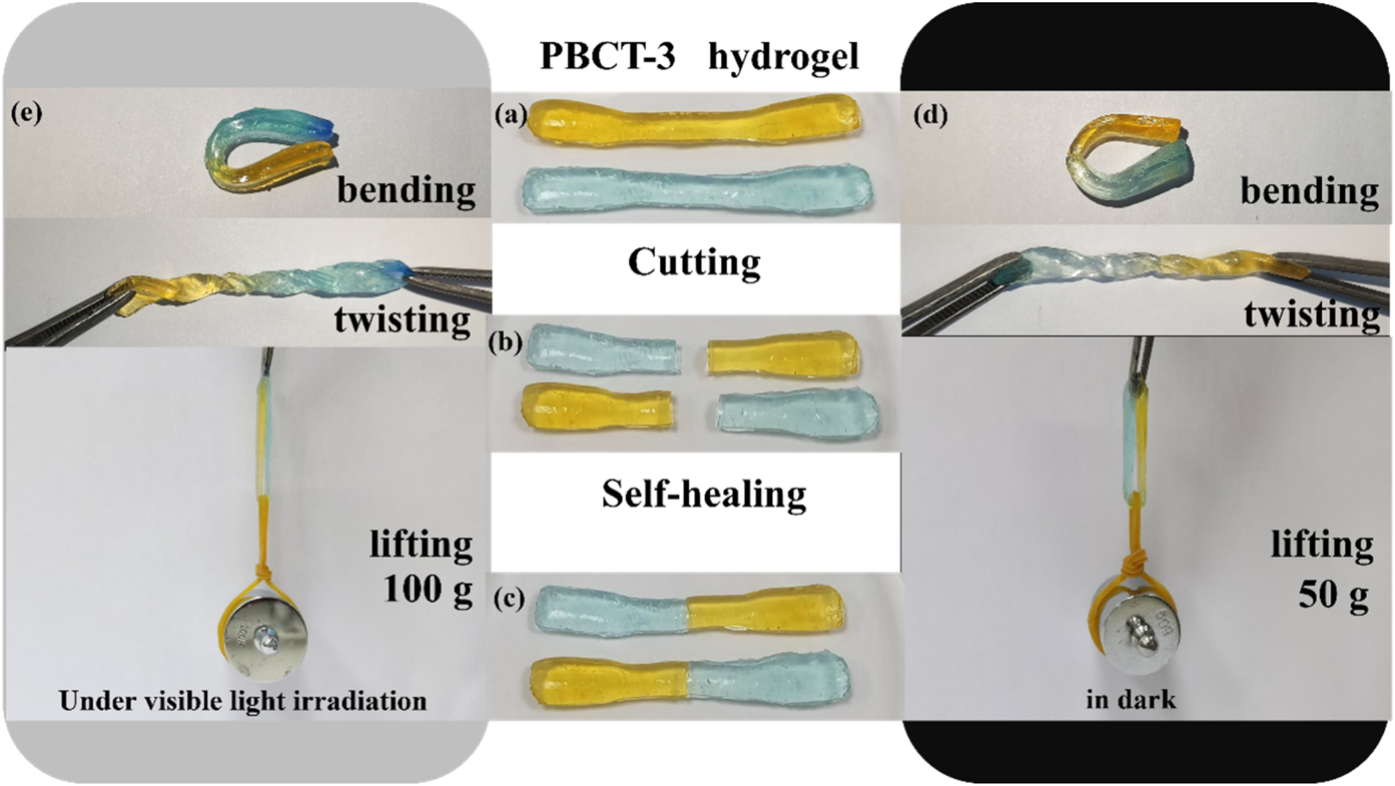
Self-healing behavior of the PBCT-3 hydrogel under visible light irradiation and in darkness.

Moreover, after self-healing, no obvious vestige of the physical damage was observed at the junction. Self-healed PBCT hydrogels can be easily bent and twisted, as shown in Fig. 8. However, if the self-healing process is accompanied by visible light irradiation, the tensile strength of the PBCT hydrogels was significantly increased from 50 g to 100 g. At the same time, the addition of the SiO_2_@g-C_3_N_4_@TiO_2_ nanoparticles also improved the strain rate of the composite hydrogels, as shown in Fig. S9.†

**Fig. 9:**
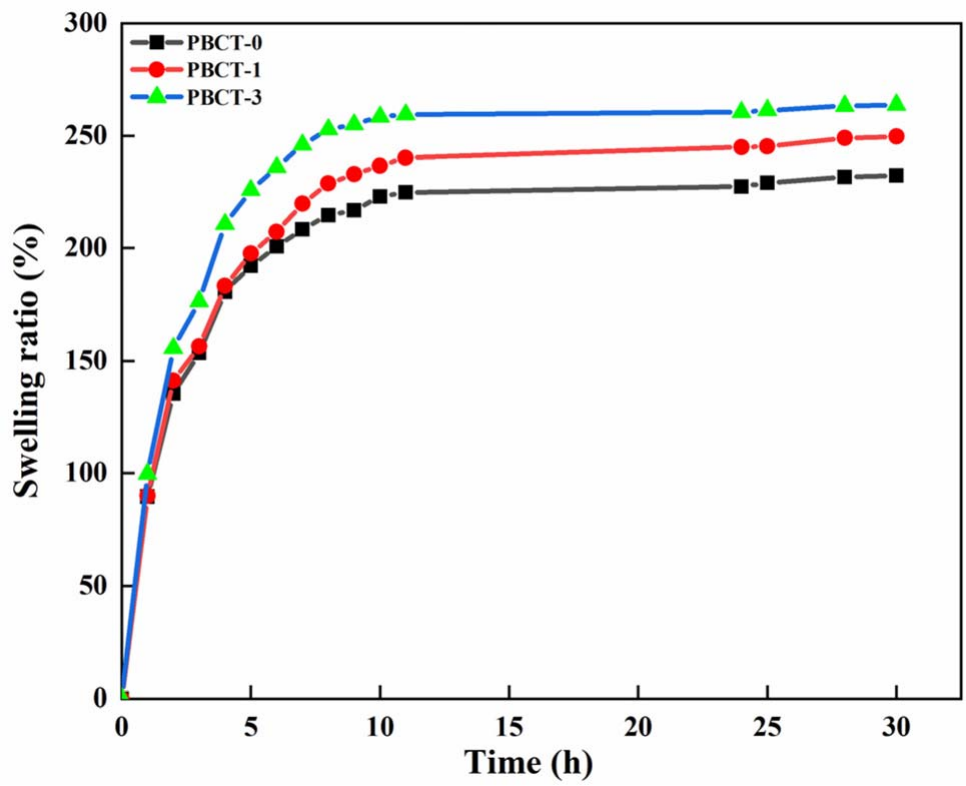
Effect of the SiO_2_@g-C_3_N_4_@TiO_2_ nanoparticles on the swelling rate of the PBCT hydrogel.

The swelling ratio of hydrogel is one of the important indicators, which reflects the volume change of hydrogels after absorbing water. Fig. 9 shows the swelling ratio of PBCT hydrogels with different amounts of the SiO_2_@g-C_3_N_4_@TiO_2_ nanoparticles. The experimental results indicated that the SiO_2_@g-C_3_N_4_@TiO_2_ nanoparticles can also enhance the swelling ratio of the PBCT hydrogels. All of the PBCT hydrogel samples reached their maximum swelling rate after 10 h. With the increase of the amount of the SiO_2_@g-C_3_N_4_@TiO_2_ nanoparticles, the swelling rate also increased accordingly. The equilibrium swelling ratio of PBCT-0 was 210%, whereas those of PBCT-1 and PBCT-3 were 228.73% and 252.91%. The phenomenon may be due to that the addition of the SiO_2_@g-C_3_N_4_@TiO_2_ nanoparticles led to the formation of more holes inside the PBCT hydrogels. However, the hydrophilicity of the SiO_2_@g-C_3_N_4_@TiO_2_ nanoparticles may also promote the swelling rate of hydrogels.

## 4. Conclusions

In summary, using polyvinyl alcohol, borax, chitosan, and a type of semiconductor composite nanoparticle of SiO_2_@g-C_3_N_4_@TiO_2_ as raw materials, we developed a novel type of photo-regulated self-healing hydrogel (PBCT) by a facile freezing–thawing method. Under visible light irradiation in the self-healing process, the self-healing and mechanical properties of the PBCT hydrogel doped with a certain trace amount of SiO_2_@g-C_3_N_4_@TiO_2_ nanoparticles were greatly improved, and the self-healing efficiency of the hydrogel increases from 26.67% to 45.67% in darkness, whereas that increased from 41.33% to 65.67% under visible light irradiation. At the same time, the addition of a trace amount of SiO_2_@g-C_3_N_4_@TiO_2_ nanoparticles also improved the swelling property of the hydrogels. This work provides a new strategy for constructing photo-regulated smart hydrogels.

## Conflicts of interest

There are no conflicts to declare.

## Supplementary Material

RA-015-D5RA01507C-s001

## Data Availability

The data that support the findings of this study are available in the article or its ESI.†
