# Supplementary material for: A novel photo-regulated self-healing hydrogel based on hollow SiO2@g-C3N4@TiO2 and PVA
Source: RSC Adv. 2025 Jun 23;15(27):21300–10. doi: 10.1039/d5ra01507c (PMC12184326; doi:10.1039/d5ra01507c)
Supplement: RA-015-D5RA01507C-s001 [file RA-015-D5RA01507C-s001.pdf]

Supplementary Information (SI) for RSC Advances.
This journal is © The Royal Society of Chemistry 2025

Supporting information

A novel kind of photo-regulated self-healing hydrogel based on hollow SiO_2@g-C_3N_4@TiO_2 and PVA

*Yu-Ling Qi, Hao-Yu Zhou, Guo-Zhi Han**

College of Chemistry and Molecular Engineering, Nanjing Tech University, Nanjing, 211816, P.R. China

Corresponding Author

Tel.: ＋86 139 5193 9398

Email: han@njtech.edu.cn

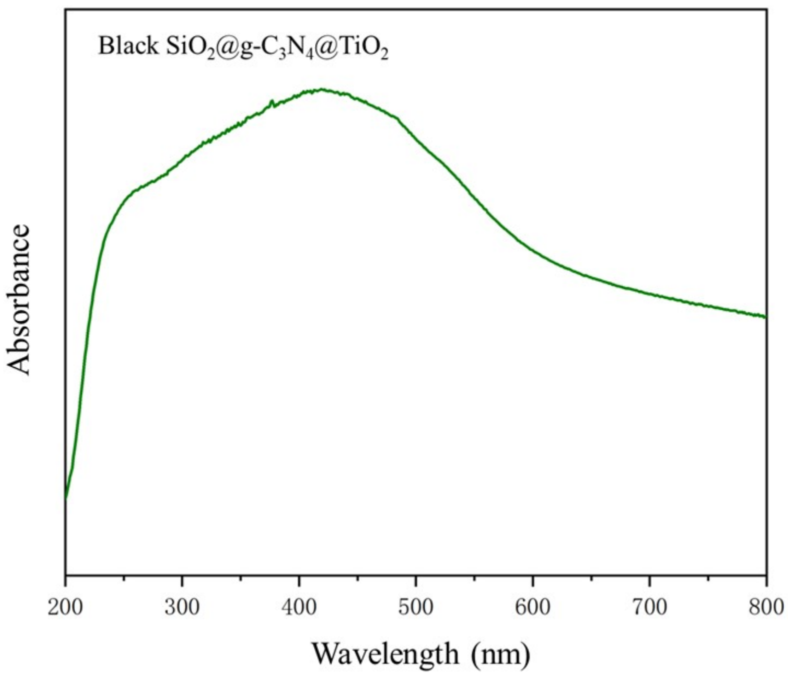

Figure S1. The UV-vis diffuse reflectance spectra of black SiO_2@g-C_3N_4@TiO_2 nanoparticles

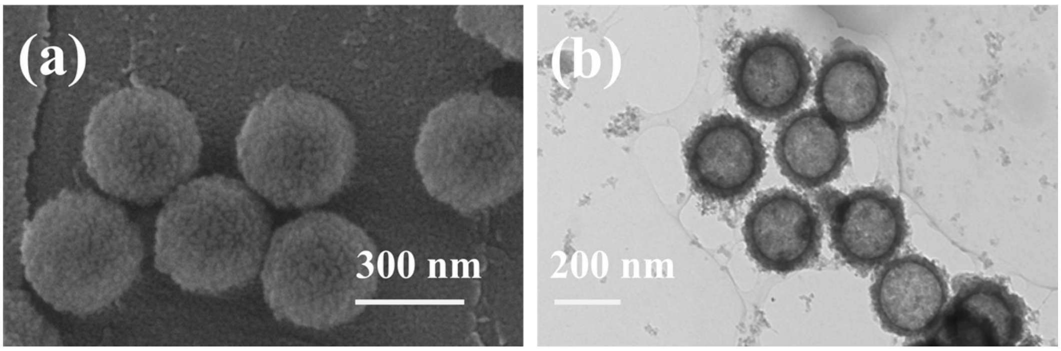

Figure S2. SEM and TEM images of the hollow SiO_2@g-C_3N_4@TiO_2 microspheres and reference samples.

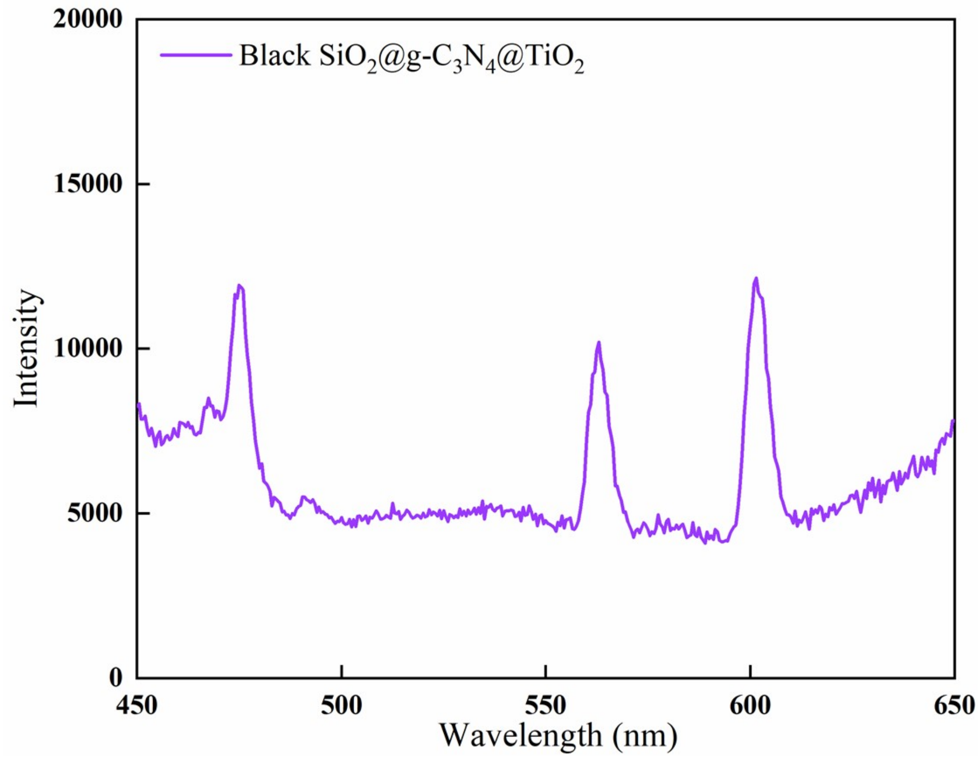

Figure S3. PL spectra of the black SiO_2@g-C_3N_4@TiO_2 nanoparticles.

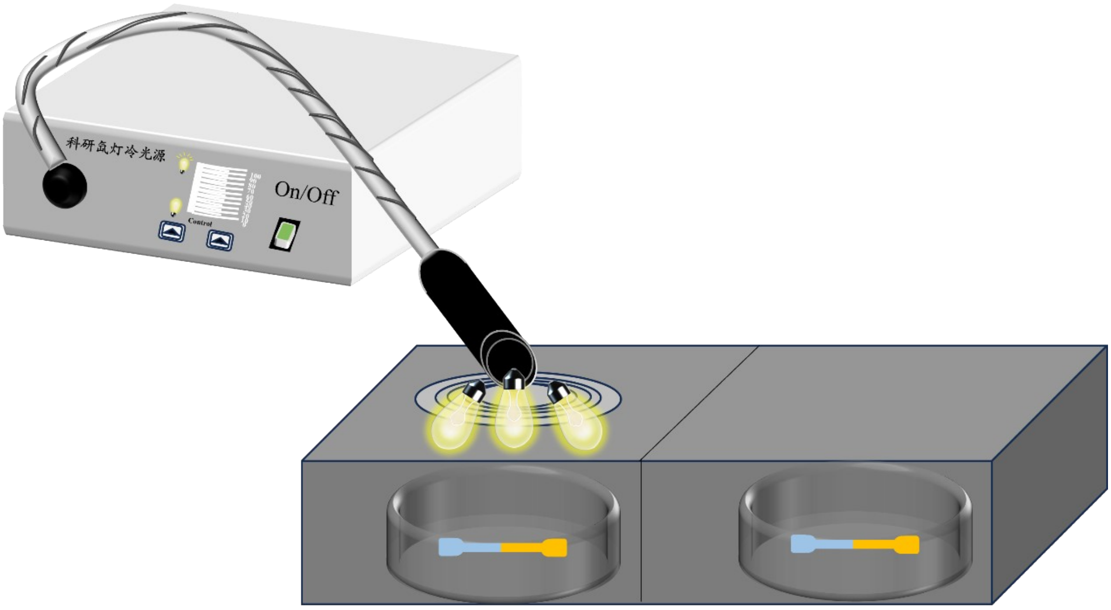

Figure S4. Test of self-healing performance of the PBCT hydrogels.

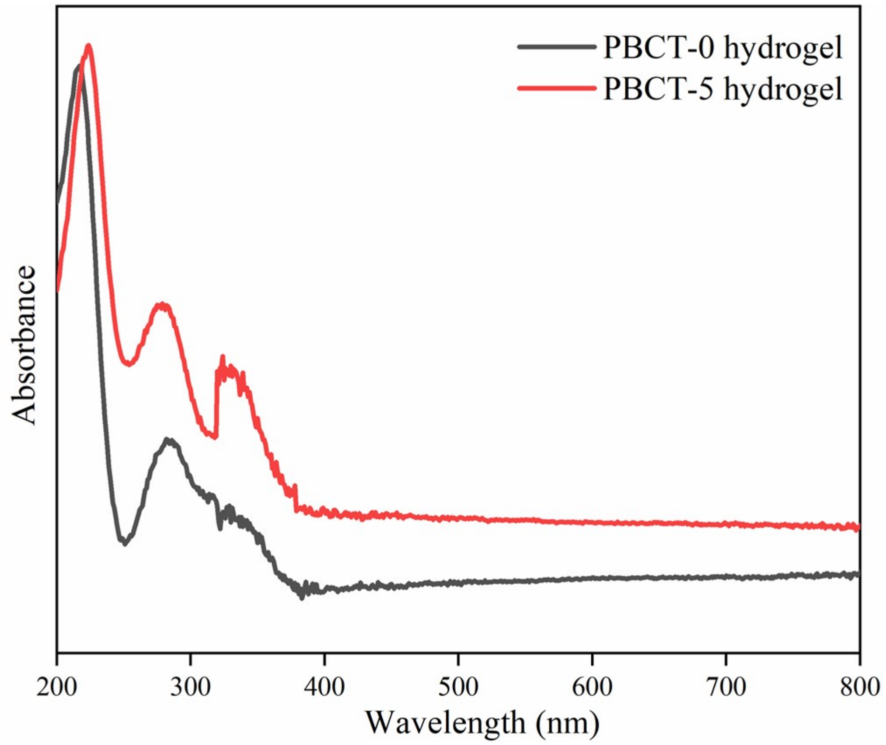

Figure S5. The UV-vis diffuse reflectance spectra of PBCT hydrogels.

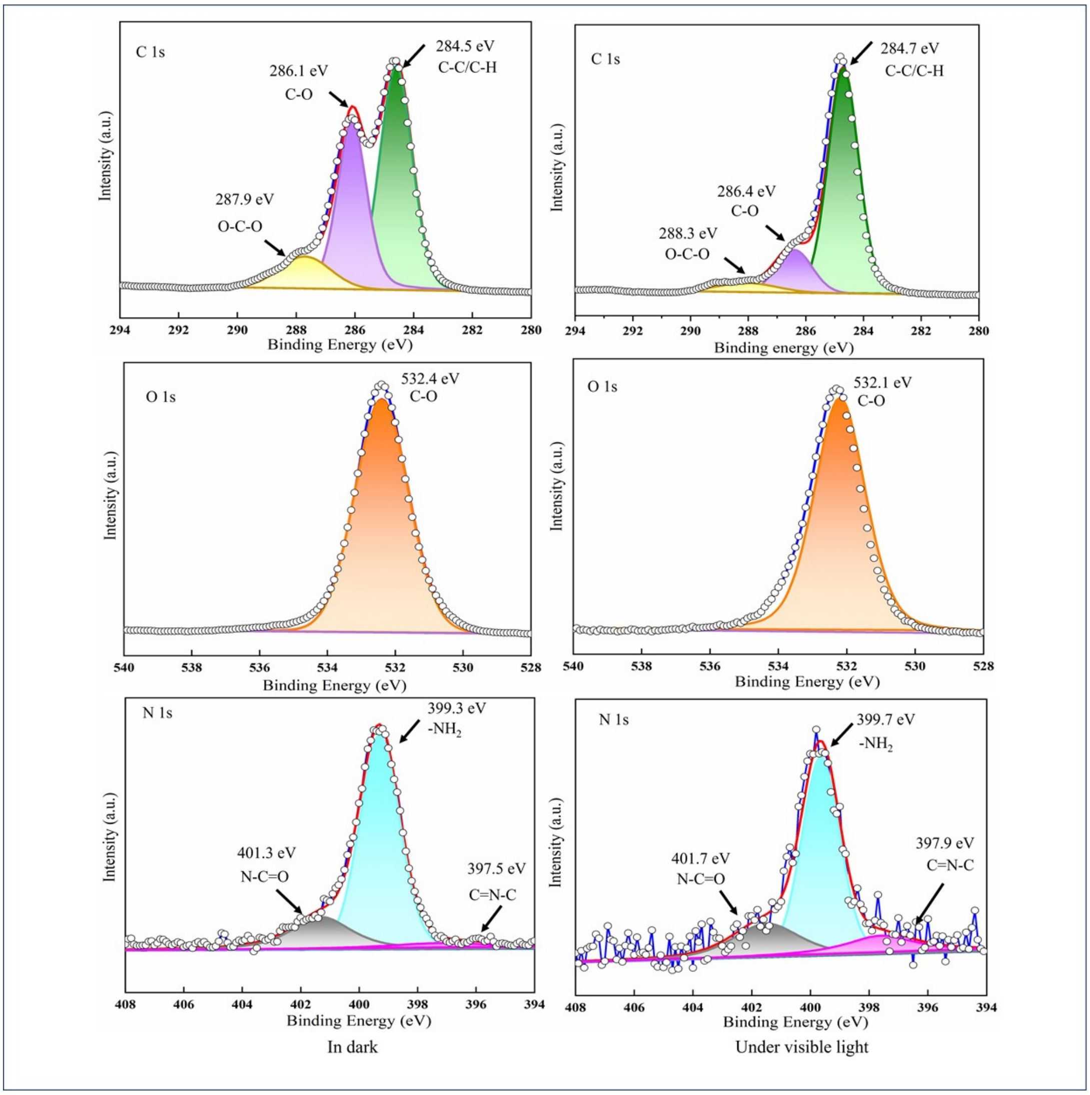

Figure S6. XPS spectra of the PBCT-3 hydrogel before visible light irradiation and after visible light irradiation.

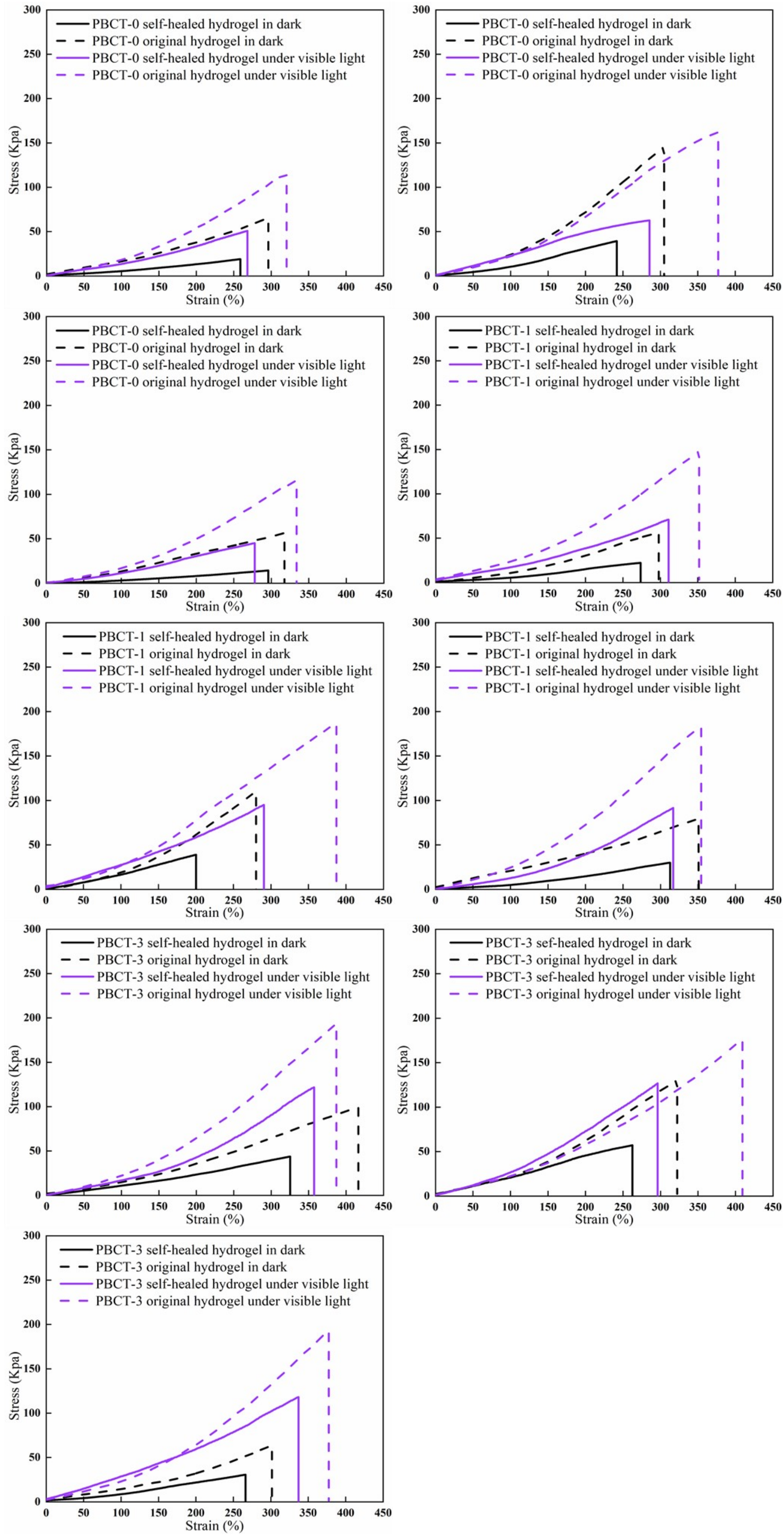
PBCT-0 self-healed hydrogel in dark
PBCT-0 original hydrogel in dark
PBCT-0 self-healed hydrogel under visible light
PBCT-0 original hydrogel under visible light
PBCT-1 self-healed hydrogel in dark
PBCT-1 original hydrogel in dark
PBCT-1 self-healed hydrogel under visible light
PBCT-1 original hydrogel under visible light
PBCT-3 self-healed hydrogel in dark
PBCT-3 original hydrogel in dark
PBCT-3 self-healed hydrogel under visible light
PBCT-3 original hydrogel under visible light
PBCT-3 sef-healed hydrogel in dark
PBCT-3 sef-healed hydrogel under visible light
Stress (Kpa)
Strain (%)

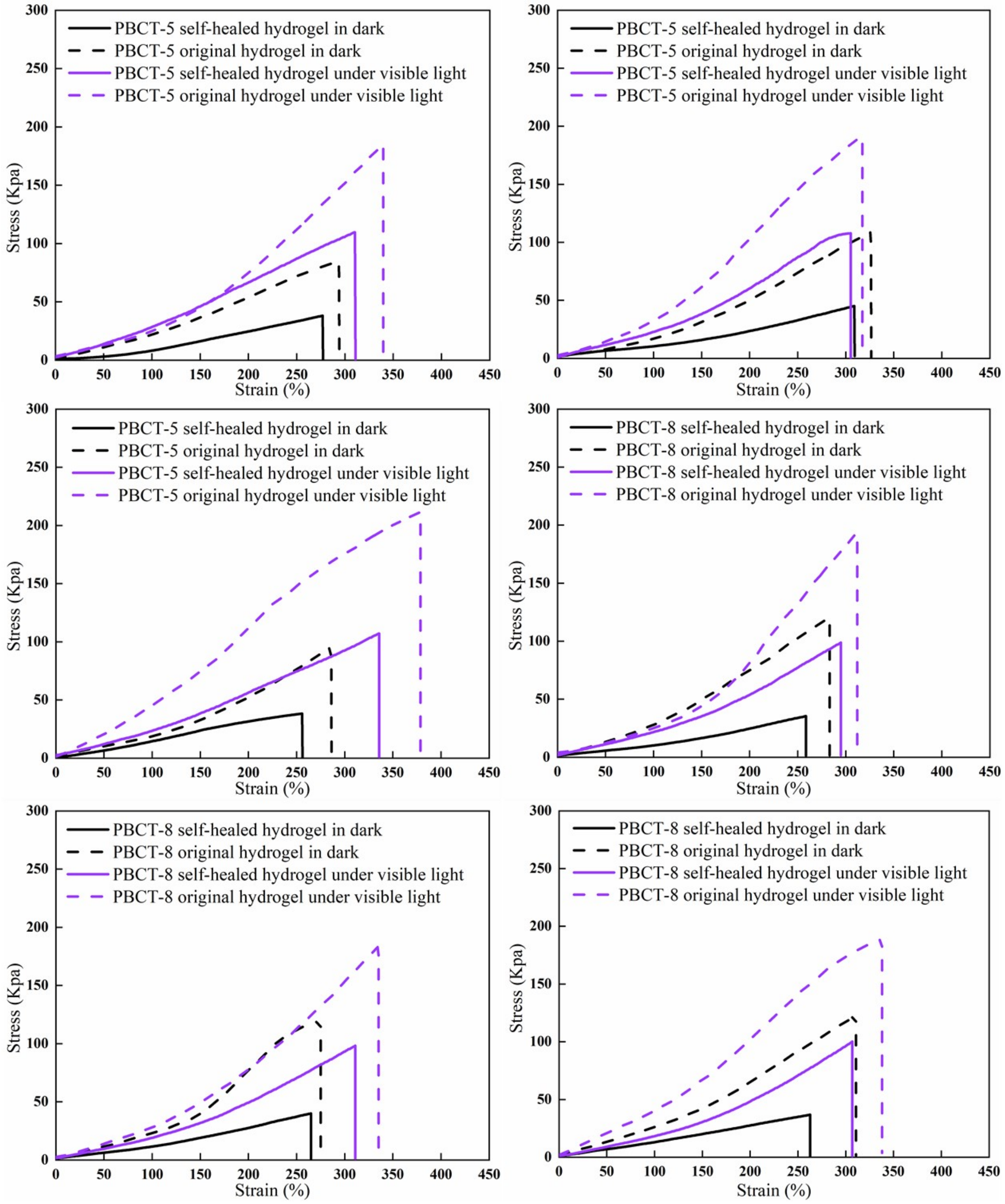

Figure S7. Stress-strain curves of the PBCT self-healed hydrogels with different amount of SiO_2@g-C_3N_4@TiO_2 nanoparticles.

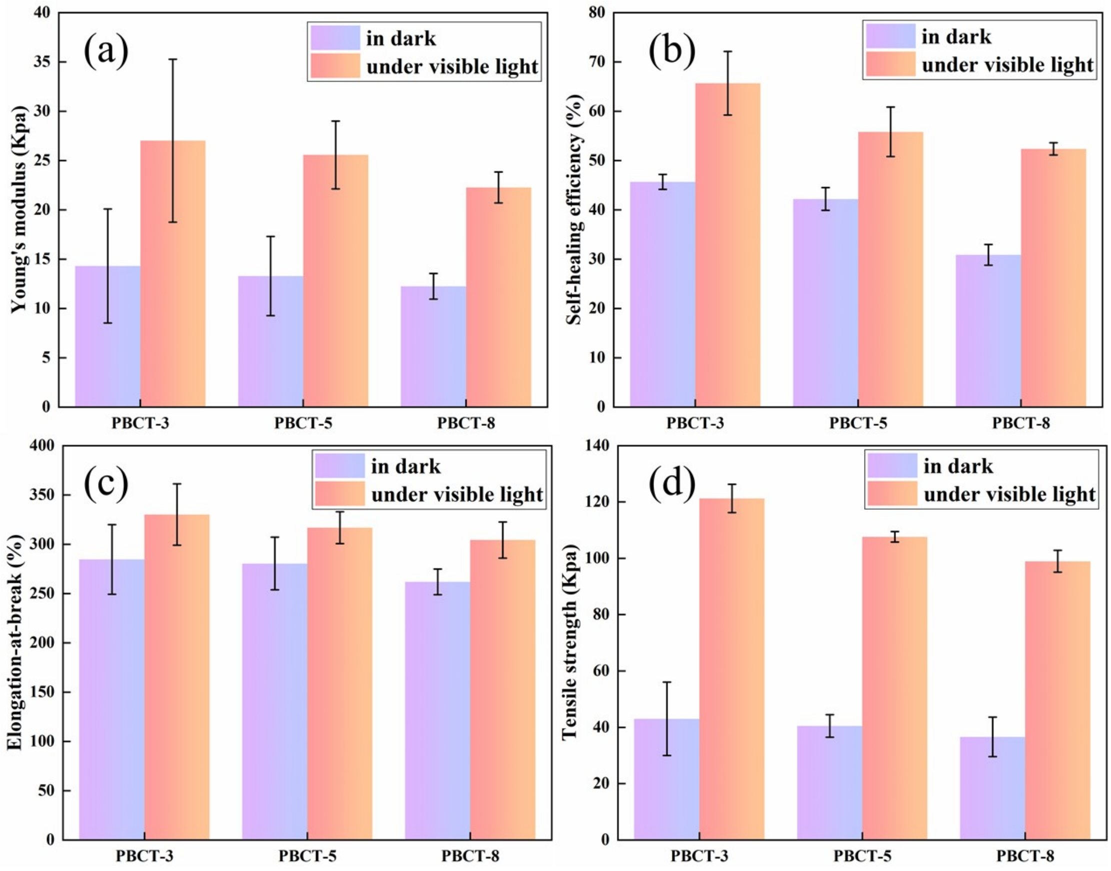

Figure S8. Effect of visible light irradiation on the self-healing of the PBCT-3, PBCT-5 and PBCT-8 samples.

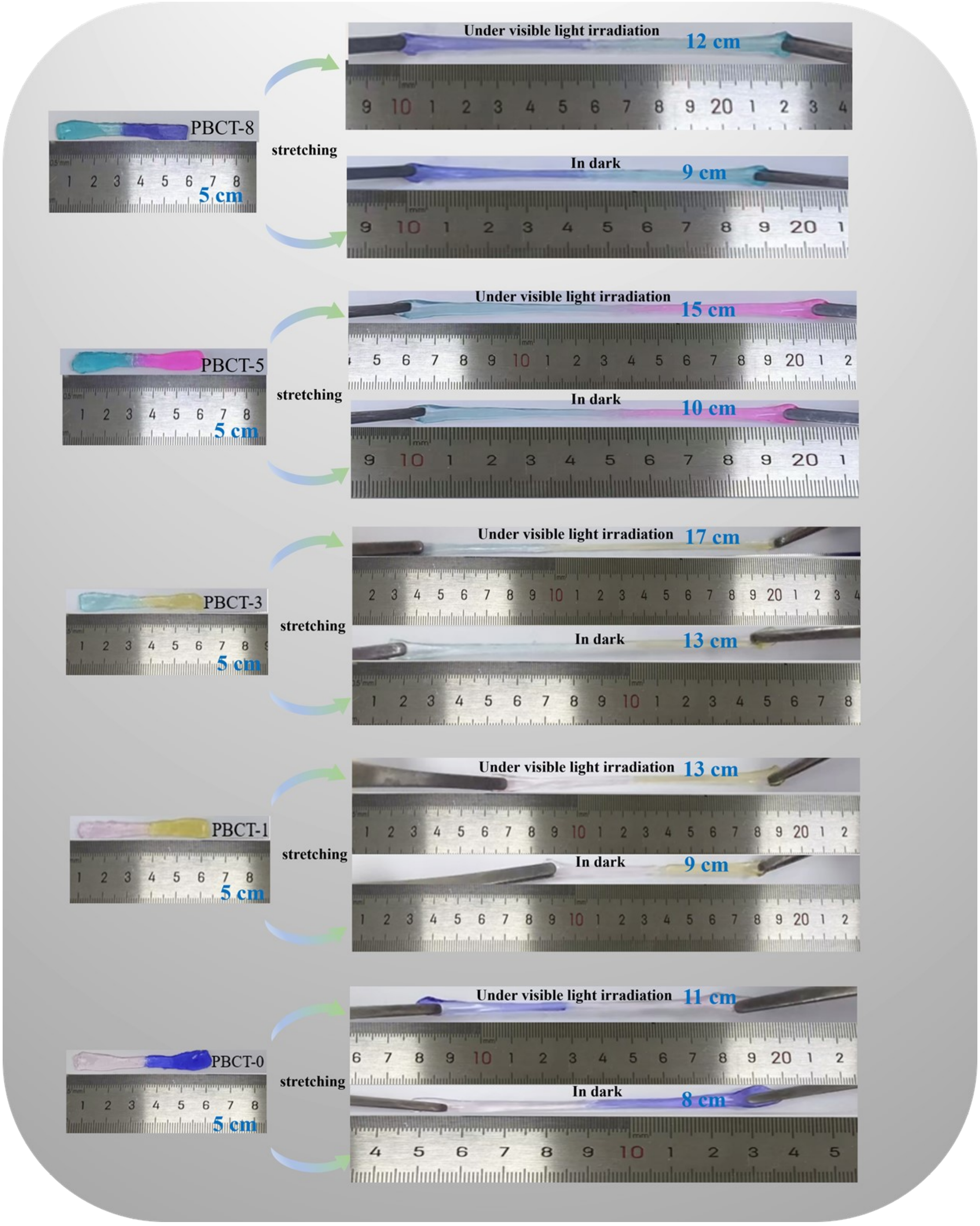

Figure S9. Photos of PBCT self-healed hydrogels for stretching in dark and under visible light irradiation respectively.
